# Functional Characterization, Mechanism, and Mode of Action of Putative Streptomycin Adenylyltransferase from *Serratia marcescens*

**DOI:** 10.3390/antibiotics11121722

**Published:** 2022-11-30

**Authors:** Dhamodharan Prabhu, Sundararaj Rajamanikandan, Mathimaran Amala, Poopandi Saritha, Jeyaraman Jeyakanthan, Palaniappan Ramasamy

**Affiliations:** 1 Research and Development Wing, Sree Balaji Medical College and Hospital, Bharath Institute of Higher Education and Research (BIHER), Chennai 600 044, Tamil Nadu, India; 2 Structural Biology and Bio-Computing Laboratory, Department of Bioinformatics, Alagappa University, Karaikudi 630 003, Tamil Nadu, India

**Keywords:** functional annotation, antibiotic resistance, streptomycin adenylyltransferase, ANT

## Abstract

Nosocomial infections are serious threats to the entire world in healthcare settings. The major causative agents of nosocomial infections are bacterial pathogens, among which *Enterobacteriaceae* family member *Serratia marcescens* plays a crucial role. It is a gram-negative opportunistic pathogen, predominantly affecting patients in intensive-care units. The presence of intrinsic genes in *S. marcescens* led to the development of resistance to antibiotics for survival. Complete scanning of the proteome, including hypothetical and partially annotated proteins, paves the way for a better understanding of potential drug targets. The targeted protein expressed in *E. coli* BL21 (DE3) pLysS cells has shown complete resistance to aminoglycoside antibiotic streptomycin (>256 MCG). The recombinant protein was purified using affinity and size-exclusion chromatography and characterized using SDS-PAGE, western blotting, and MALDI-TOF analysis. Free phosphate bound to malachite green was detected at 620 nm, evident of the conversion of adenosine triphosphate to adenosine monophosphate during the adenylation process. Similarly, in the chromatographic assay, adenylated streptomycin absorbed at 260 nm in AKTA (FPLC), confirming the enzyme-catalyzed adenylation of streptomycin. Further, the adenylated product of streptomycin was confirmed through HPLC and mass spectrometry analysis. In conclusion, our characterization studies identified the partially annotated hypothetical protein as streptomycin adenylyltransferase.

## 1. Introduction

“Nosocomial” or “Healthcare-Associated Infections (HAI)” are widely used to refer to any class of disease affecting patients while undergoing medical care or even sometimes after treatment procedures. Prolonged stay in hospitals is found to be the root cause of HAI, and its risk factors range from simple to critical health infections, leading up to fatalities [1]. A recent study reported that these HAIs are rigorously intensifying in primary infections, even leading to deaths, with developing countries bearing 75% of the burden of infection-associated mortality, especially in neonates [2]. HAIs have become unavoidable complications in medical procedures due to (i) aging, (ii) prolonged stay of immune compromised patients in hospitals, (iii) rapid advancements in invasive devices-assisted diagnosis, and (iv) inappropriate usage of antimicrobial agents [3]. The causative agents of HAIs are microbes viz. bacteria, protozoa, fungi, viruses, and mycobacteria, but 90% of these infections are caused by bacteria [4]. *Staphylococcus aureus*, *Acinetobacter* spp., *Pseudomonas aeruginosa*, *Streptococcus* spp., and *Enterobacteriaceae* family members including *Klebsiella pneumoniae*, *Proteus mirablis*, *Escherichia coli*, and *Serratia marcescens* are widely reported to be the bacterial species causing HAIs [1].

Gram-negative facultative anaerobe *S. marcescens* is a notorious pathogen belonging to the *Enterobacteriaceae* family. It is responsible for intravenous catheter-associated infections, urinary tract infections, blood-stream endocarditis, and septicemia in humans. The prevalence of *S. marcescens* infections is high among intensive care unit patients [5]. Due to the presence of several virulence factors and misuse of antibiotics, the control and treatment of *S. marcescens* infections has become difficult. *S. marcescens* carries the intrinsic genes in addition to plasmid-encoding genetic elements to develop resistance against all classes of antibiotics; it rapidly acquires resistance even to the newly developed antimicrobials [6]. It is reported that *S. marcescens* apply different resistance mechanisms such as (i) antibiotics modification or degradation, (ii) ribosome site alteration, (iii) increasing efflux activity or decreasing permeability, and (iv) modification of metabolic pathways for survival in the antibiotic environment [7]. In *S. marcescens,* inactivation of aminoglycoside antibiotics through structural modification catalyzed by aminoglycoside-modifying enzymes (AMEs) has been found to be the preferred resistance mechanism [8]. Aminoglycoside antibiotics have always been the first choice of treatment against bacterial infections and have shown broad-spectrum activity [9]. The amino group in streptomycin provides a positive charge to the entire molecule, which facilitates the binding of streptomycin into the negatively charged cleft (A site) of the 30 s ribosome, which in turn leads to the inhibition of bacterial protein synthesis. Binding of streptomycin in the tRNA acceptor A site of the 30 s ribosome causes a misreading and hinders the tRNA translocation step in protein translation [10]. Clinically, AMEs play a pivotal role in driving resistance to streptomycin. Streptomycin adenylyltransferase (SMATase) catalyze the transfer of the adenyl group from the ATP molecule to the specific hydroxyl group in streptomycinin, a process referred to as adenylation. Aminoglycoside o-nucleotidyl transferases share very little sequence identity; however, they retain similar ATP and Mg^2+^ binding folds essentially required for catalytic activity [11].

Antibiotic resistance has become a global threat and has been a serious problem to handle in recent years [12]. Worldwide complete genomes of various organisms have been sequenced through different genome projects that have piled up large sets of information to be explored. However, only a limited number of gene products have been annotated, while a significant fraction of data has been marked as hypothetical [13]. In this study, a computationally annotated protein based on sequence similarity was experimentally characterized to understand its complete function. The observations of the present study provide deeper knowledge on the mode of action of the protein, and this information could aid in the design of novel lead molecules that synergistically combat antibiotic resistance and *S. marcescens* infection when administered with appropriate antibiotics.

## 2. Materials and Methods

### 2.1. Cloning and Transformation

The targeted gene (EMBL-EBI ID: AGB84093) from *S. marcescens* was computationally predicted to be aminoglycoside adenylyltransferase based on sequence homology [14]. The targeted gene was cloned in a pET-28a (+) vector using restriction enzymes NdeI and XhoI under the control of alac promoter purchased from GenScript. The recombinant plasmid was transformed into *Escherichia coli* BL21 (DE3) pLysS cells (Agilent Technologies) by heat-shock method. The pET-28a (+) vector possesses the kanamycin resistance gene and the *E. coli* BL21 (DE3) pLysS cells themselves carry the chloramphenicol resistance gene. The transformed bacterial (*E. coli* BL21 (DE3) pLysS) cells were cultured on Luria Bertani (LB) agar plate containing 50 µg/mL kanamycin and 34 µg/mL chloramphenicol overnight at 37 °C. The positive colonies from the plate were selected and inoculated into fresh LB liquid medium and allowed to grow for 16 h at 37 °C. The cells were then used for recombinant plasmid isolation. Plasmid was performed using the Qiagen DNA Mini-Prep kit, and the plasmids were sequenced to confirm the site of insertion, orientation, and integrity.

### 2.2. Expression and Purification of Recombinant Protein

Recombinant cells containing the plasmid were grown in 100 mL LB liquid medium containing 50 μg/mL kanamycin and 34 μg/mL chloramphenicol at 37 °C until the OD_600_ reached 0.4 to 0.6. Subsequently, different concentrations of isopropylβ-D-1-thiogalacto-pyranoside (IPTG) (0.1, 0.3, 0.5, and 1.0 mM) was added to induce the expression of recombinant protein with optimum expression at 18 °C. The induced cells were then harvested after 20 h of incubation, and the cells were lysed by sonication. Protein expression profiling was performed by sodium-dodecyl-sulphate–polyacrylamide gel electrophoresis (SDS-PAGE). The overnight bacterial culture was inoculated (1% inoculum) into 4 L LB broth containing 50 μg/mL kanamycin and 34 μg/mL chloramphenicol and again allowed to grow at 37 °C until OD_600_ reached around 0.4 to 0.6. Subsequently, the temperature was brought down to 16 °C before the induction of cells for recombinant protein expression with 1 mM IPTG. The cells were collectively grown for a period of 24 h. The harvested cells were centrifuged at 19,802× *g* for 20 min to separate LB medium from cells and the pellet obtained was resuspended in Tris buffer (50 mM Tris, 350 mM sodium chloride (NaCl), 5% Glycerol, 10 mM Imidazole, 3 mM β-mercaptoethanol) at pH 6.8 for cell lysis. Protease cocktail inhibitor was added to the resuspended cell pellet in addition to lysozyme and thoroughly mixed for 20 min at 4 °C. Deoxyribonuclease (DNase) and 5 mM magnesium dichloride (MgCl_2_) were added to the lysate mixture and stirred for 20 min at 4 °C before the pulse-field sonication process (5-sec pulse on and 10-sec pulse off). The sonicated blend was centrifuged at 19,802× *g* for 30 min, and the supernatant was loaded into a pre-equilibrated HisTrap™ HP 5 mL column (GE Healthcare). The column was washed with five column volumes of buffer containing 50 mM Tris, 350 mM NaCl, 3 mM β-mercaptoethanol (BME), 5% glycerol, and 10 mM imidazole to remove unbound proteins. Then, 250 mM imidazole in the elution buffer eluted the His-tagged proteins bound to the column. All elutes corresponding to the targeted protein were concentrated and applied on a pre-equilibrated HiLoad 16/60 Superdex 75 gel-filtration column. The collected fractions were analyzed on SDS-PAGE and concentrated to 12 mg/mL using an Amicon concentrator (10 kDa).

### 2.3. Protein Confirmation by Western Blotting and MALDI Analysis

The elutes collected from His-Trap affinity chromatography and HiLoad 16/60 Superdex 75 gel-filtration chromatography were analyzed using 12% SDS-PAGE for protein profiles. The gels were stained using Coomassie brilliant blue G-250 dyes for visualization. In western blotting, the electrophoresis gel was transferred to a nitrocellulose membrane to detect the specific proteins by antibody tagging [15]. Upon transfer of the protein samples to the membrane, it was blocked using Tris buffer saline (TBS) containing 5% skimmed milk powder. After 2 h of incubation, the membrane was washed with 0.1% of Tween 20 and incubated with anti-His tag antibody produced in rabbit (primary antibodies, Sigma-Aldrich, St. Louis, MO, USA) for 2 h. The membrane was washed again with 0.1% of Tween 20 to remove excessive primary antibodies. Later, anti-mouse IgG (whole molecule alkaline phosphatase antibody produced in rabbit (1:5000 dilution) secondary antibodies, Sigma-Aldrich) were added and incubated for 1 h. The unbound secondary antibodies were washed with 0.1% of Tween 20. Developer solution was prepared by dissolving 5 mg of nitro nlue tetrazolium (NBT) and 6.25 mg of 5 bromo-4-chloro, 3-indolyl-phosphate (BCIP) in 500 µL and 125 µL of alkaline phosphate buffer (pH 9.5), respectively. Finally, the developer solution was added to the nitrocellulose membrane and incubated in the dark for 15–30 min to visualize the protein bound to specific antibodies. The targeted protein was carefully excised from the gel, was finely chopped into pieces, and was de-stined using 100 µL of 50% acetonitrile (ACN) dissolved in 50 mM ammonium bicarbonate. The mixture was incubated at room temperature for 15 min, and the de-staining process was repeated until the gel turned colorless [16]. The protein in the mixture was reduced with 10 mM dithiothreitol (DTT) and 100 mM ammonium bicarbonate for 15 min at room temperature before centrifugation. The pellet was treated with 50 mM iodoacetamide dissolved in 100 mM ammonium bicarbonate for alkylation. Then, 20 µL of sequencing-grade trypsin (10 units) was incubated with the centrifuged mixture at 37 °C for 16 h. The trypsin-digested mixture was treated with 5% TriFluoro acetic acid (TFA) in 50% ACN and centrifuged. The supernatant containing the peptides was stored at −20 °C. Mass spectrometry analysis was conducted using matrix-assisted laser desorption/ionization time-of-flight/time-of-flight (MALDI TOF/TOF) (UltrafleXtreme MALDI TOF/TOF, Bruker Daltonics GmbH, Bremen, Germany) at the Proteomics Facility, IISc, Bangalore. A solution of CHCA (α-cyano-4-hydroxy trans-cinnamic acid) at 10 mg/mL was used as a MALDI matrix. The charged ions were measured by employing MALDI-TOF/TOF fitted with a 337 nm nitrogen laser. The spectra were acquired in the range of 700–4500 Da, and the peaks were analyzed with the query in the Mascot database.

### 2.4. Antibiotic Susceptibility Test and Determination of MIC

To understand the functional role of the target protein in the recombinant cells, an antibiotic susceptibility test based on Bauer and Kirby’s disk diffusion method was employed [17]. Overnight-grown bacterial cultures were swabbed on LB agar plates containing 50 μg/mL kanamycin and 34 μg/mL chloramphenicol in the presence of 1 mM IPTG. Discs coated with 19 antibiotics at specific concentrations were placed on the solidified agar plates and incubated overnight at 35 °C for susceptibility tests. The antibiotics coated in the discs diffused through the LB agar plates, resulting in the formation of a zone of inhibition [18]. The recombinant cells were assayed for the determination of minimum inhibitory concentration (MIC) using various antibiotics such as amikacin, ampicillin, erythromycin, gentamicin, kanamycin, streptomycin, and tobramycin. Cultures of recombinant *E. coli* pLysS cells that were grown overnight were swabbed onto LB agar plates containing 50 μg/mL kanamycin, 34 μg/mL chloramphenicol, and 1 mM IPTG. Antibiotics-coated gradient Ezy MIC™ Strips (HiMedia) of amikacin, ampicillin, erythromycin, gentamycin, kanamycin, streptomycin, and tobramycin were applied to recombinant *E. coli* BL21 pLysS cells swabbed ino Petri plates and incubated for 16 h at 37 °C [19]. The Ezy MIC™ Strips were coated with specific antibiotics on a gradient scale from 0.16 µg/mL to 256 µg/mL. MICs were measured at the lowest concentration of antibiotics, where no bacterial growth was visible. Similarly, empty *E. coli* BL21 pLysS cells (cell control) and *E. coli* BL21 pLysS cells with empty pET28a+ (vector control) were also grown overnight tested for antibiotic susceptibility and MIC.

### 2.5. Malachite Green Assay

Malachite green (MG) assay is a colorimetric-based detection method used to quantify the amount of free organic phosphate released in a reaction [20]. The reaction buffer was composed of 30 mM tris hydrochloric acid, pH 7.5, 140 mM NaCl, 40 mM MgCl_2_, 1 mM DTT, 200 µM adenosine triphosphate (ATP), 200 µM pyrophosphatase, and 200 µM streptomycin. Two different concentrations of protein samples were used for this study along with positive control (sodium phosphate). To adjust the linear time for the assays, 10 mM ethylenediaminetetraacetic acid (EDTA) was used to stop the reaction. MG solution was prepared by slowly adding 60 mL of concentrated sulphuric acid into 300 mL of water. The mixture was kept in ice to reduce the heat liberated by the concentrated acid. Then, 0.44 g of MG was dissolved in this solution, turning it into a yellow color. This solution is long-term stable at room temperature (master solution). The final working MG solution was prepared by mixing 2.5 mL of 7.5% ammonium molybdate in 10 mL of the master solution along with 0.2 mL of 11% Tween 20 at the time of use. MG solution was added in a ratio of 1:4 (1 volume of MG solution and 4 volumes of the reaction mixture) to detect the phosphates. The assay was performed in a 96-well plate, and the absorbance of the reaction mixture with MG solution was measured at 620 nm at various time intervals. All the reactions were performed in triplicate, and the mean values were used for analysis.

### 2.6. Chromatographic Adenylation Assay

The reaction mixture consisted of 50 mM HEPES (pH 8.0) containing 2 mM streptomycin, 10 mM ATP, 160 mM NaCl, 10 mM MgCl_2_, and 1 mM DTT. Freshly purified protein and pyrophosphatase were added to the reaction mixture at four 6 h intervals during the 24 h incubation at room temperature with controlled agitation. The final concentration of the protein and pyrophosphatase at the end of 24 h was 0.03 mg/mL and 1.5 units, respectively. The reaction mixture containing protein was removed using a 10 Kilo Dalton (kDa) cutoff concentrator (Merck). The 5 mL of filtrate containing adenylated streptomycin was applied to a 1 mL HiTrap SP Sepharose Fast Flow column (GE Healthcare), and the unbound antibiotics, non-reacted ATPs, and adenosine monophosphates (AMPs) were removed by washing with MES buffer (20 mM MES pH 6.0). The strongly bound adenylated streptomycin was eluted using 20 column volumes of elution buffer (20 mM MES pH 6.0, 1 M NaCl) in a linear gradient manner, and the absorbance was monitored at 260 nm [21].

### 2.7. HPLC and Mass Spectrometry Analysis of Adenylated Streptomycin

The 5 µL fraction of adenylated streptomycin was injected into Agilent Poroshell 120 EC-C18 (2.7 um 100 mm × 2.1 mm) in Agilent 1260 Infinity LC System. Formic acid/water (0.1% (*v*/*v*)) was used as buffer A and 0.1% (*v*/*v*) formic acid/acetonitrile as buffer B for the mobile phase. Further, the fractionated samples (100 µL) were mixed with 200 µL of methanol and incubated at −20 °C for 30 min before centrifugation at 15,000× *g*. After centrifuging for 10 min, the supernatant was used for mass spectrometry analysis in AB Sciex Instruments QTRAP 5500, which uses the Turbo V Ion Source for ionization. The standards for streptomycin (MW: 728.69), adenosine triphosphate (MW: 551.14), and adenosine monophosphate (MW: 365.24) (Sigma-Aldrich, St. Louis, MO, USA) were dissolved in 50% methanol to a concentration of 5 µg/mL for the high-performance liquid chromatography (HPLC) and mass spectrometry analyses. In mass spectrometric analysis, ATP and AMP are capable of generating signals in both positive and negative ion modes. However, the signal intensity in negative ion mode is stronger than that in positive ion mode, and streptomycin can only be detected in positive ion mode. Hence, a positive ion mode scan is commonly used to detect ATP, AMP, and streptomycin.

## 3. Results and Discussion

### 3.1. Transformation of Recombinant Plasmid

The pET28a+ vector containing the targeted gene was commercially purchased from GenScript and was transformed into *E. coli* BL21 (DE3) pLysS cells. The white color colonies observed after 16 h of incubation indicated that the cells were successfully transformed with desired plasmids. The plasmid extracted from the overnight grown cultures was sequenced (Eurofins India, Nantes, France) to confirm the ligations of the targeted gene into the vector. Sequencing results showed 100% homology with the reference sequence and confirmed the insertion and proper orientation of the targeted gene into the vector.

### 3.2. Expression and Purification

**Figure 1:**
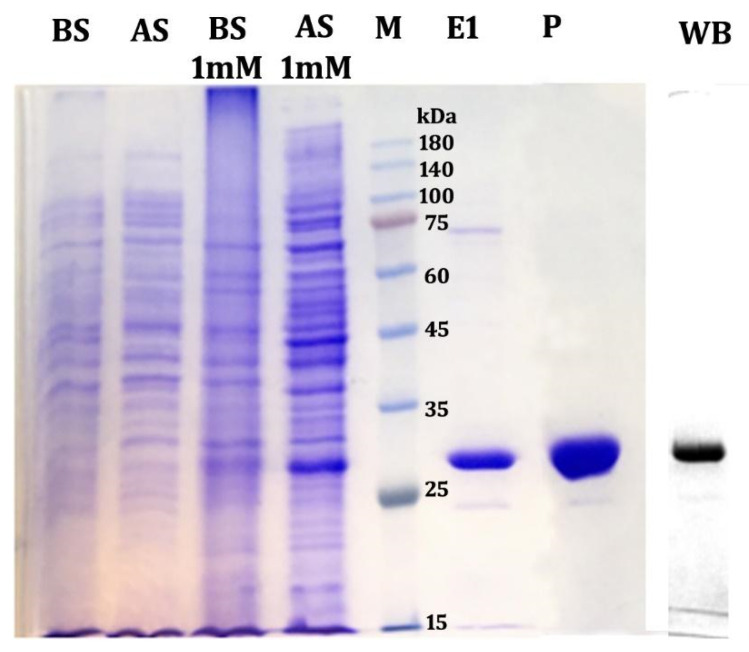
Profile of protein expression in *E. coli* BL21 (DE3) pLysS cells with and without IPTG. Lane 1: BS—Without IPTG, Before Sonication; Lane 2: AS—Without IPTG, After Sonication; Lane 3: 1 mM IPTG, Before Sonication; Lane 4: 1 mM IPTG, After Sonication; Lane 5: M—Marker; Lane 6: E1—Elute from HisTag affinity chromatography; Lane 7: P—Purified protein from size exclusion chromatography. Final lane: WB—western blot of the purified protein.

The transformed *E. coli* BL21 (DE3) pLysS cells were grown in the absence and presence of IPTG at selected temperatures for the expression of recombinant proteins. SDS-PAGE analysis of total cell extract showed the protein bands at the specific molecular weight ~30 kDa (His-tagged recombinant protein molecular weight: 29.93 kDa) (Figure 1). Maximum expression of recombinant protein was observed at 1 mM concentration of IPTG, whereas 0.1, 0.3, and 0.5 mM concentrations showed less expression of recombinant protein. No expression was identified in the IPTG deficient sample, which confirms the tight control of recombinant protein expression under the T7 promoter. For large-scale production, the cells were grown in LB medium containing 50 μg/mL kanamycin and 34 μg/mL chloramphenicol at 37 °C until OD reached 0.4 to 0.6. Computed GRAVY value of 0.037 depicted the hydrophilic nature of the protein and its solubility in polar solvents [22]. Cell growth at low temperature reduce the rate of protein synthesis, which provides proper time for protein folding and thus promotes the conversion of protein into a soluble fraction [23]. The recombinant protein was purified with nickel-based affinity chromatography, which selectively trapped the histidine tags. Based on the protein sequence, the target protein was predicted to have an isoelectric point of 4.90 and a theoretical molecular weight of 29113.4 Da [24]. The fractions collected were analyzed in SDS-PAGE and revealed a band corresponding to ~30 kDa in 12% gel (Figure 1). The concentrated protein samples of collective fractions were injected into the size exclusion column, resulting in the separation of proteins based on molecular weight. The purified recombinant protein appeared as a homogenous clear band in 12% SDS-PAGE (Figure 1), indicating that the purified recombinant protein was ~99% pure.

### 3.3. Identification of Purified Protein by Western Blotting and MALDI

To confirm the purified recombinant protein, western blotting and MALDI TOF/TOF mass spectrometry analysis were performed. The anti-his antibody was used to recognize hexahistidine repeats in the purified recombinant protein. Due to the absence of hexahistidine tag, anti-his antibodies do not recognize other host cell proteins. The western blot showed a positive band at molecular weight ~30 kDa, corresponding with the recombinant protein (Figure 1) and clearly confirming that the targeted protein was purified. Further, mass data search of the trypsin-digested fragments in the Mascot database identified the presence of similar mass peptide fragments in four proteins. Out of these four proteins, the two peptides (56APLDNTQR63 and 175ETADLQGDER184) of the targeted protein sequence matched with the aminoglycoside adenylyltransferase from *S. marcescens* (Score: 70 (Table 1).

### 3.4. Functional Characterization

The hypothetical proteins in *S. marcescens* FGI94 were computationally analyzed, and these functions of the proteins were predicted based on the sequence [14] and structural information [22]. Investigation of the hypothetical proteins including partially annotated proteins would lead to the identification of novel targets or new mechanistic insights towards the development of potential drug molecules [25]. The peptides of the targeted protein indicated that the protein functions as an aminoglycoside adenylyltransferase. Experimental characterization of proteins is essential to deduce their complete function [26].

The antibiotic susceptibility test results for 19 antibiotics are shown in Supplementary Materials Table S1. All three sample groups (Cell Control, Vector Control, and Recombinant Cell) showed a high level of sensitivity to 17 antibiotics. A high level of chloramphenicol resistance was observed due to the presence of the chloramphenicol resistance gene in *E. coli* BL21 (DE3) pLysS cells [27]. Complete resistance to the antibiotic kanamycin was seen only in the vector control cells and recombinant cells, whereas sensitivity was observed in cell control. Similar to chloramphenicol, resistance to kanamycin was seen due to the presence of the *kanR* gene in the pET28a vector [28]. 

Complete streptomycin resistance was observed in the recombinant cells, while cell control and vector control cells showed high sensitivity to streptomycin. Due to the presence of aminoglycoside adenylyltransferase enzyme, *Salmonella enteric* has been reported to show a similar resistance to the antibiotic streptomycin [21]. This confirms that the expression of the targeted protein in recombinant cells is responsible for the resistance against the aminoglycoside antibiotic streptomycin in *E. coli* BL21 (DE3) pLysS cells. MICs of selected antibiotics against the cell control, vector control, and recombinant cells were determined using HiMedia gradient concentration Ezy MIC™ Strips and are tabulated in Table 2. In the presence of targeted protein, cells were highly resistant to streptomycin (up to 256 µg/mL); however, sensitivity was observed against amikacin, ampicillin, erythromycin, gentamycin, and tobramycin. Similarly, empty *E. coli* BL21 pLysS cells (cell control) and *E. coli* BL21 pLysS cells containing empty pET28a+ vectors were found to be sensitive against all tested antibiotics. Based on the antibiotic susceptibility and MIC results, it is evident that the recombinant protein is involved in the modification activity that leads to streptomycin resistance. The streptomycin resistance induced by the target protein indicates that the protein mediates resistance through the inactivation of antibiotics by the adenylation process [22] and functions as streptomycin adenylyltransferase (SMATase). A sequence-based evolutionary study has shown a wide distribution of SMATase in gram-positive and gram-negative organisms. Similarly, multiple sequence analysis has revealed a high degree of sequence similarity within the aminoglycoside adenylyltransferase family and has shown that variations in the catalytic site determine substrate specificity [22]. Streptomycin adenylyltransferase of S. *marcescens* was homologous and possessed 54% sequence similarity to the aminoglycoside adenylyltransferase of *Salmonella enterica*. Structural analysis of aminoglycoside adenylyltransferase family has revealed two distinct domains; the negatively charged inter-domain cleft traps the aminoglycoside antibiotics [21,24,29].

### 3.5. Malachite Green Assay

**Figure 2:**
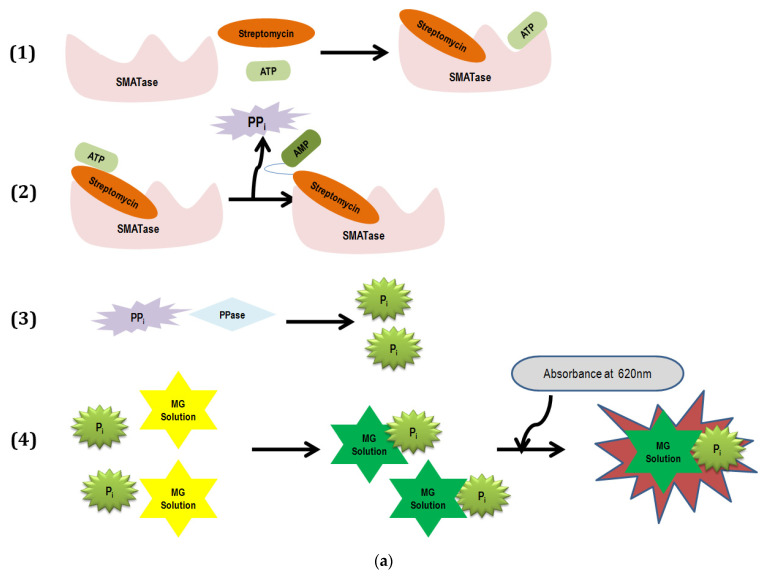

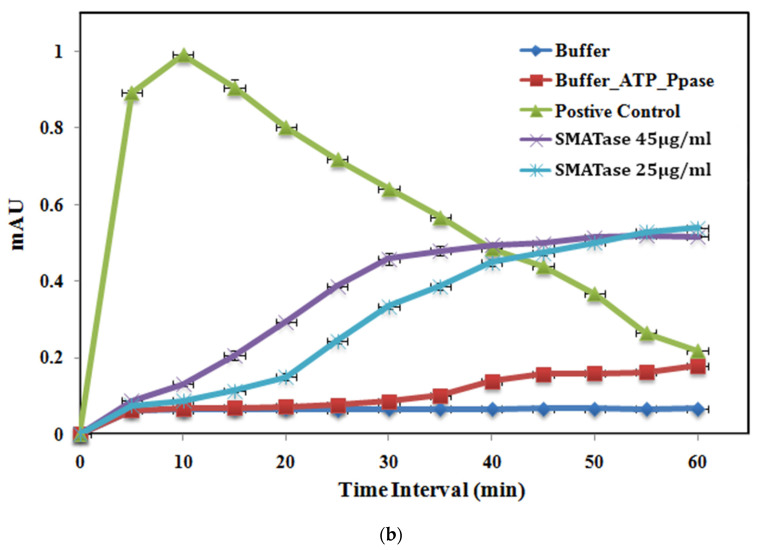
(**a**) Schematic stepwise working principle of malachite green adenylation assay. (**b**) Malachite green adenylation assay: detection of free phosphate liberated from the adenylation process is captured by malachite green solution at 620 nm. Standard phosphate of 0.5 M was used as positive control, and two different concentrations of enzymes (45 µg/mL and 25 µg/mL) were used to determine the enzyme.

The enzymatic property of SMATase was detected by MG assay, quantifying the number of pyrophosphates liberated from the adenylation reaction. The enzyme pyrophosphatase (PPase) catalyzes the irreversible hydrolysis of the phosphoanhydride bond to produce two phosphates (Pi) from inorganic pyrophosphate (PPi). The free phosphates (Pi) were captured by the MG molybdate complex, which turns the yellow color solution into green and shows an absorbance peak at 620 n, compared to MG absorbance at 446 nm (Figure 2a). The standard phosphate solution (0.5 M) was used in the reaction as a positive control to evaluate the experimental setup. Mixtures of different compositions were analyzed to determine the enzymatic activity, and the absorbance of the MG molybdate complex recorded is shown in Figure 2b. The enzymatic activity graph depicts that the free phosphates were only detected in the presence of SMATase and reveals that the amount of pyrophosphate released from ATP was purely dependent on the SMATase concentration. A significant increase in activity was recorded at higher concentrations of SMATase, whereas no changes in absorbance were observed in the absence of SMATase. Standard phosphate solution showed higher absorbance in the initial time points due to the availability of free phosphates, which were eventually trapped by the MG solution. Subsequently, the absorbance gradually decreased with the depletion of free phosphates in the standard phosphate solution. The absorbance values are directly proportional to the number of pyrophosphates released from the adenylation process and in turn record the approximate rate of enzyme activity. The results show evidence of the process of adenylation occurring in the presence of SMATase and ATP by releasing pyrophosphates. Streptomycin was reported to have a similar adenylation activity on *Salmonella enterica*’s aminoglycoside adenylyltransferase while having no effect in its absence [21].

### 3.6. Chromatographic Adenylation Assay

**Figure 3:**
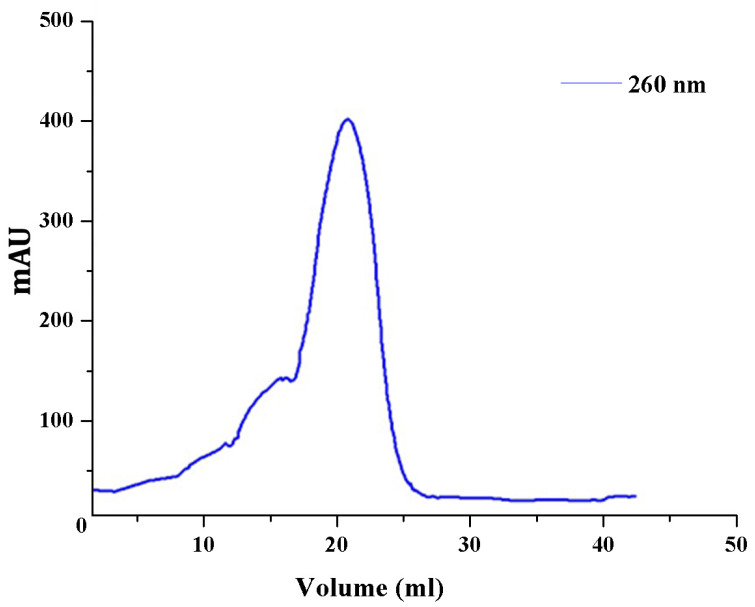
Chromatographic adenylation assay: adenylated streptomycin retains the positive charge even after the attachment of adenyl group from donor ATP are absorbed at 260 nm in GE ÄKTA™ (FPLC).

A cation-exchange chromatographic adenylation assay developed by Stern AL, 2018 was consecutively performed to confirm the streptomycin adenylation process. Either the phosphate released from the adenylation of streptomycin or unproductive ATP hydrolysis was directly detected in this assay [21] (Figure 3). HiTrap SP Sepharose Fast Flow column containing negatively charged immobilized resins tends to attract the positively charged molecules in the reaction mixture. The unmodified ATP and AMP carry negative charges and may not be able to bind in the column due to the charge similarity. The unmodified streptomycin was bound to the column due to its positive charge and not absorbed at 260 nm, whereas nucleotides and nucleotide substitutions alone were absorbed. Streptomycin, even after adenylation, carries a positive charge and absorbs at 260 nm due to the presence of the adenyl group. After passing 20–25 mL of high salt concentration buffer into the column by linear gradient, the positively charged adenylated streptomycin bound to column was detected at 260 nm (Figure 3). Adenyltransferase enzymes, similar to polymerases, make use of a two-metal-ion mechanism for the catalytic process [21,29,30]. The adenyl transfer process carried out by aminoglycoside adenylyltransferase is identical to the reactions carried out by two-metal-ion polymerases and nucleases that have been thoroughly studied and have active sites with very similar arrangements of residues. Similar to the adenylated associative mechanisms, the catalytic residue Glu-87 was predicted to play a prominent role in the adenylation process, which is predicted to extract a proton from the hydroxyl group of streptomycin to enable the oxygen to facilitate the nucleophilic attack on the α-phosphate of ATP [21,31].

### 3.7. Confirmation of Adenylated Streptomycin by HPLC and Mass Spectrometry

**Figure 4:**
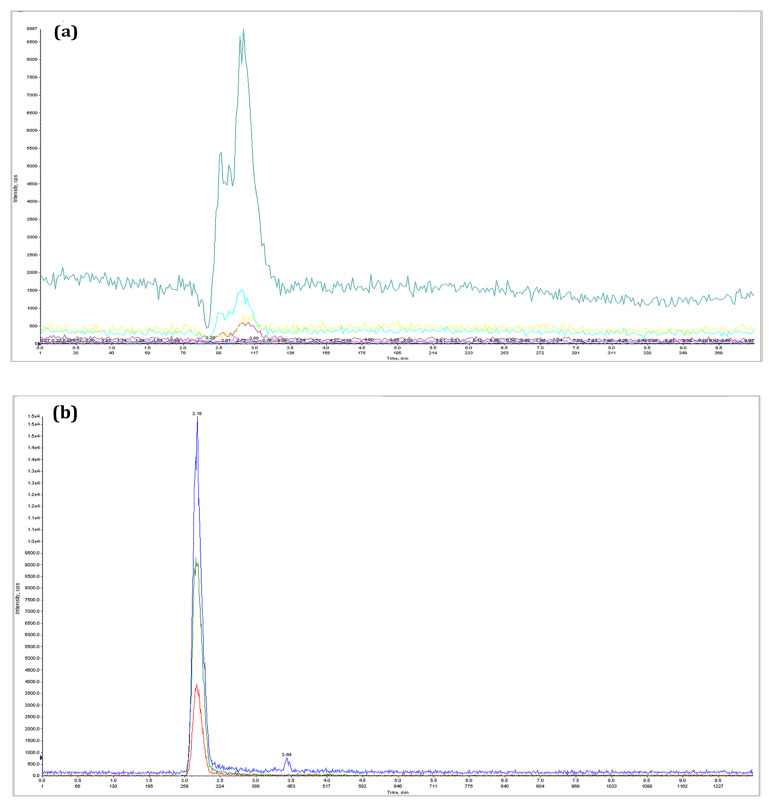

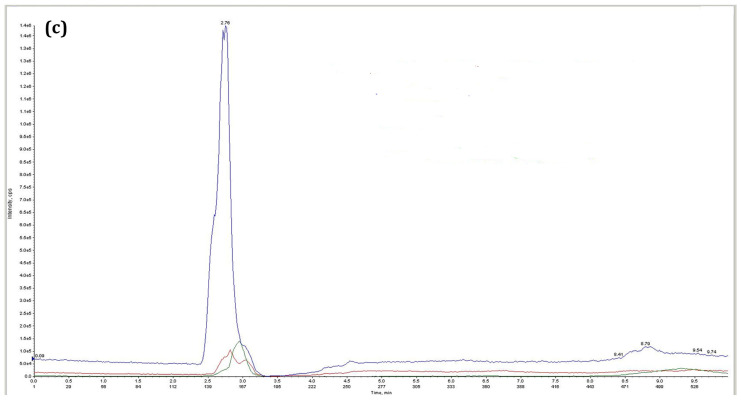
Retention analysis in HPLC: (**a**) elution chromatogram of ATP; time interval: 2.3 to 3.3. (**b**) Elution chromatogram of streptomycin; time interval: 2.0 to 2.50. (**c**). Elution chromatogram of adenylated streptomycin; time interval: 2.40 to 3.30.

**Figure 5:**
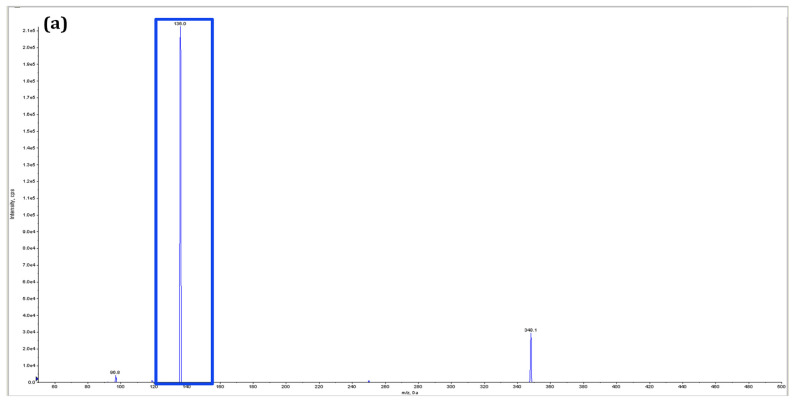

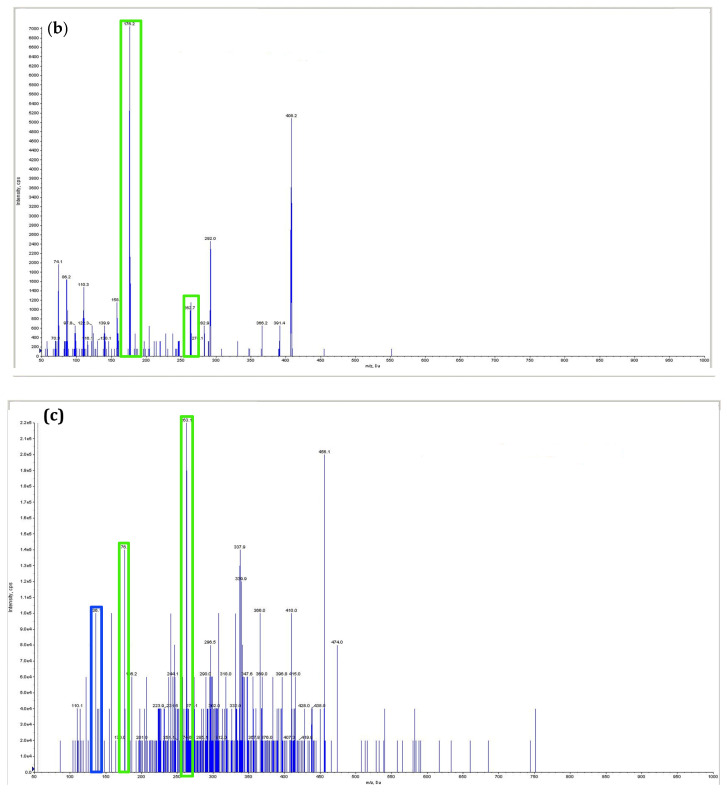
Mass spectrometry analysis: (**a**) MS/MS spectrum of AMP; adenine peak *m*/*z* at 136.2 is highlighted in blue color. (**b**) MS/MS spectrum of streptomycin; two peaks *m*/*z* at 263.2 and 176.2 are highlighted in green color (**c**) MS/MS spectrum of adenylated streptomycin. The stronger spectrum of *m*/*z* at 136.2 from AMP and *m*/*z* at 263.2 and 176.2 from streptomycin, commonly observed in adenylated streptomycin, is highlighted in blue and green color, respectively.

Chromatographic analysis revealed that the elution behavior of standard ATP and streptomycin are similar. From Figure 4, it is clear that the elution time of ATP and streptomycin are 2.3–3.3 and 2.0–2.50 min, respectively. The elution time of putative adenylated streptomycin was recorded between 2.40 to 3.30 min. Clearly, the elution time was distinct from the elution times of ATP and streptomycin. Hence, it confirmed that the product was adenylated streptomycin. Figure 5 shows the mass spectrums of the standard ATP, AMP, streptomycin, and adenylated streptomycin. The *m*/*z* value of ATP is 508.3, but the signal intensity with two sodium ions *m*/*z* of 552.2 is stronger. The MS/MS result showed that in addition to the peak of the original precursor ion *m*/*z* = 508.2, the fragments with stronger signals were *m*/*z* 410.2 and 136.2. The *m*/*z* value of streptomycin is 582.3, but the signal with two hydrogen ions [M + 2H] *m*/*z* of 292.2 is stronger. The MS/MS result showed that in addition to the peak of the original parent ion *m*/*z* = 582.2, the fragments with stronger signal intensity were *m*/*z* 408.2 and 176.2. The *m*/*z* value of AMP is 348.2. The MS/MS result showed that the fragments with stronger signal intensity were *m*/*z* 97.2 and 136.2. The putative adenylated streptomycin molecular weight is theoretically calculated as 912.2. Concordantly, a mass spectrometric scan on the putative adenylated streptomycin samples resulted in the identification of 912.2 as the molecular weight. Two peaks, *m*/*z* = 176.2, 263.2 of standard streptomycin were observed in adenylated streptomycin. Similarly, the common adenine peak *m*/*z* = 136.2 was observed in standard AMP and putative adenylated streptomycin. The common structural mass functionalities observed in the spectrometric analysis among the AMP, streptomycin, and putative adenylated streptomycin confirm the presence of adenine moiety in the streptomycin. Further, the control chromatographic adenylation reactions carried out in the absence of streptomycin and enzymes failed to produce any signals similar to streptomycin and adenylated streptomycin in MS analysis. Based on the experimental results of the in vitro MIC determination, adenylation assays, HPLC, and MS analysis, the targeted protein function is completely characterized as streptomycin adenylyltransferase, which transfers the adenyl group from ATP to streptomycin for inactivation to develop resistance.

## 4. Conclusions

The partially annotated gene cloned into pET28a+ vector was successfully transformed and expressed for purification of the protein. Affinity and gel filtration chromatography methods were employed to obtain 99% pure protein. The purified recombinant protein was confirmed using western blotting and MALDI-TOF/TOF mass spectrometry analysis. The targeted protein expressing cells showed the MIC >256MCG to the antibiotic streptomycin. The functional role of the protein in adenylation of streptomycin was confirmed by MG and chromatographic adenylation assays. Biochemical assay characterized the function of the targeted protein as streptomycin adenylyltransferase and its mechanism mediating the resistance to antibiotics. The novel inhibitors targeting SMATase could thus enhance the streptomycin activity. Synergistic activity of potential inhibitors in combination with streptomycin are expected to produce superfluous action in hampering the protein translation process. Exploring the binding of streptomycin, ATP, and MgCl_2_ would provide more insights into understanding the process of resistance in detail, which will be more useful in designing inhibitor molecules. Our findings provide a basic understanding of the protein, and further biophysical analysis coupled with structural studies will provide more insights into how streptomycin adenylyltransferase may help to overcome antibiotic resistance.

## Figures and Tables

**Table 1:** Protein identification: MALDI-TOF/TOF mass spectrometry analysis data of trypsin-digested peptides of the targeted protein were matched against data in the Mascot database. Mass information of two peptides of the targeted protein showed a Mascot score of 70.

**General Information**					
Database			NCBIpro		
Score			70		
Monoisotopic mass (Mr)			29,095		
Calculated pI			4.93		
Theoretical mass of protein			29,936		
Matches			3		
Matching protein			Aminoglycoside nucleotidyltransferase		
Matching source organism			*Serratia* sp. FGI94		
**Peptide Information**					
Peptide fragment		56APLDNTQR63		175ETADLQGDER184	
Score		59		67	
Mr (Expt)		913.4564		1132.4988	
Mr (Calc)		913.4981		1132.4996	
Observed		914.4636		567.2567	

**Table 2:** Antibiotic susceptibility and minimal inhibitory concentration (MIC) tests: seven antibiotics in discs and Ezy MIC™ strips were tested against the cell control, vector control, and recombinant cell. Cell control (CC), empty *E. coli* pLysS cell; vector control (VC), *E. coli* pLysS cell with null pET28a vector; recombinant cell (SM), *E. coli* pLysS cell bearing recombinant vector expressing SMATase protein. Tested against the empty *E. coli* pLysS cell (cell control), *E. coli* pLysS cells bearing empty pET28a(+) vector (vector control) and recombinant *E. coli* pLysS cell bearing target gene expressing SMATase (recombinant cell).

	MIC (µg/mL)					
Antibiotic	*E. coli* pLysS Cells (Cell Control)		*E. coli* pLysS Cells + Null pET28a(+) (Vector Control)		*E. coli* pLysS Cells + SMATase—pET28a(+) (Recombinant Cell)	
	Disc Diffusion (ZOI in mm)	Ezy MIC™ Strips (MIC in µg/mL)	Disc Diffusion (ZOI in mm)	Ezy MIC™ Strips (MIC in µg/mL)	Disc Diffusion (ZOI in mm)	Ezy MIC™ Strips (MIC in µg/mL)
Amikacin	28	0.75	31	0.75	31	0.75
Ampicillin	29	1	33	1	33	1
Erythromycin	10	16	10	16	10	16
Gentamycin	29	0.25	24	0.25	32	0.25
Kanamycin	26	0.75	-	No inhibition #	-	No inhibition #
Streptomycin	28	3	29	3	-	No inhibition *
Tobramycin	29	0.50	34	0.50	34	0.50

ZOI—zone of inhibition as per CLSI interpretative chart; *—complete resistance observed up to 256 µg/mL; **#**—presence of kanR selection marker in pET28a(+) vector resulted in no significant inhibition up to ~128 µg/mL; non-detectable or minimal inhibition of cells around the strips in the range of ~128–256 µg/mL was visible.

## Data Availability

All data generated and analyzed during this study are included in this article.

## Supplementary Materials

The following are available online at https://www.mdpi.com/article/10.3390/antibiotics11121722/s1, Table S1: Antibiotic Susceptibility Test: 19 antibiotics tested against the empty *E. coli* pLysS cell (Cell Control), *E. coli* pLysS cells bearing empty pET28a vector (Vector Control) and recombinant *E. coli* pLysS cell bearing target gene expressing SMATase (Recombinant Cell).
