# Supplementary material for: Functional Characterization, Mechanism, and Mode of Action of Putative Streptomycin Adenylyltransferase from Serratia marcescens
Source: Antibiotics (Basel). 2022 Nov 30;11(12):1722. doi: 10.3390/antibiotics11121722 (PMC9774460; doi:10.3390/antibiotics11121722)
Supplement: Supplementary file 1 [file antibiotics-11-01722-s001.zip › antibiotics-1972994-supplementary.pdf]

antibiotics

Supplementary Materials

Functional Characterization, Mechanism and Mode of Action of the Putative Streptomycin Adenylyltransferase from *Serratia marcescens*

Table S1. Antibiotic Susceptibility Test: 19 antibiotics tested against the empty E. coli pLysS cell (Cell Control), E. coli pLysS cells bearing empty pET28a vector (Vector Control) and recombinant E. coli pLysS cell bearing target gene expressing SMATase (Recombinant Cell).

Antibiotic	Code	Cell Control	Vector Control	Recombinant Cell
		Zone of Inhibition		
Penicillin-G	P	18 mm	22 mm	18 mm
**Kanamycin**	**K**	**26 mm**	–	–
Ciprofloxacin	CIP	40 mm	37 mm	40 mm
Carbenicillin	CB	33 mm	32 mm	32 mm
Ampicillin	AMP	29 mm	33 mm	33 mm
**Chloramphenicol**	**C**	–	–	–
Tobramycin	TOB	29 mm	34 mm	34 mm
Erythromycin	E	10 mm	10 mm	10 mm
Nalidixic Acid	NA	31 mm	33 mm	31 mm
Tetracycline	TE	28 mm	25 mm	29 mm
Norfloxacin	NX	38 mm	32 mm	40 mm
Rifampicin	RIF	12 mm	12 mm	12 mm
Ofloxacin	OF	38 mm	33 mm	40 mm
Gentamycin	GEN	29 mm	24 mm	32 mm
Imipenem	IPM	37 mm	30 mm	30 mm
Levofloxacin	LE	36 mm	33 mm	31 mm
**Streptomycin**	**S**	**28 mm**	**29 mm**	–
Amikacin	AK	28 mm	31 mm	31 mm
Vancomycin	VA	10 mm	10 mm	10 mm
